# Magnetic resonance spectroscopy in cervical spondylotic myelopathy: a systematic review of metabolite changes and clinical correlations

**DOI:** 10.3389/fmed.2025.1525218

**Published:** 2025-02-17

**Authors:** Iris Tatiana Montes-González, Dylan Paul Griswold, Fernando Peralta-Pizza, José Alberto Israel-Romero, Juan Felipe Mier-García, José Antonio Soriano-Sanchez

**Affiliations:** ^1^Clínica Imbanaco, Cali, Valle del Cauca, Colombia; ^2^Clínica Sebastián de Belalcázar, Cali, Valle del Cauca, Colombia; ^3^Clínica Cali, Cali, Valle del Cauca, Colombia; ^4^Neurological Center, ABC Campus Santa Fe Medical Center, Mexico City, Mexico; ^5^Stanford School of Medicine, Stanford, CA, United States; ^6^Department of Clinical Neurosciences, University of Cambridge, Cambridge, United Kingdom; ^7^Clínica Colombia, Cali, Valle del Cauca, Colombia; ^8^Hospital Departamental Tomás Uribe Uribe, Tuluá, Valle del Cauca, Colombia; ^9^Clínica de Occidente, Cali, Valle del Cauca, Colombia

**Keywords:** cervical spondylotic myelopathy, cervical myelopathy, spectroscopy, spinal cord, systematic review

## Abstract

**Introduction:**

Cervical spondylotic myelopathy (CSM) is a common degenerative condition characterized by narrowing of the cervical spinal canal, leading to progressive spinal cord injury and functional decline. While magnetic resonance imaging (MRI) is the gold standard for diagnosing CSM, it has limitations in predicting clinical outcomes. Magnetic resonance spectroscopy (MRS) offers metabolic insights that may enhance diagnostic and prognostic capabilities in CSM.

**Methods:**

We conducted a systematic review following the PRISMA guidelines. Comprehensive literature searches were performed in PubMed, OVID, EMBASE, Web of Science, and the Cochrane Central Register of Controlled Trials up to June 2023. Studies included human subjects with CSM, a cohort of at least 10 patients, and reported primary data on cervical spine MRS findings correlated with clinical scales such as the modified Japanese Orthopaedic Association (mJOA) scale, both pre- and post-operatively.

**Results:**

Six prospective studies involving 123 patients (average age 45.8 to 63 years) met the inclusion criteria. Common symptoms were neck pain, radicular upper-limb pain, paresthesia, and motor impairment. MRS findings indicated that symptomatic CSM patients had reduced N-acetyl aspartate to creatine (NAA/Cr) ratios and elevated choline to creatine (Cho/Cr) and choline to NAA (Cho/NAA) ratios compared to healthy controls. Lactate peaks were detected in a significant proportion of symptomatic patients, suggesting hypoxic or inflammatory injury. Decreased NAA/Cr and increased Cho/NAA ratios correlated with lower mJOA scores, indicating more severe myelopathy. Post-operative increases in NAA/Cr ratios and decreases in Cho/NAA ratios were associated with improved mJOA scores, highlighting the prognostic value of these metabolites.

**Conclusion:**

MRS provides valuable metabolic information correlating with clinical severity and functional outcomes in CSM. Reduced NAA/Cr and elevated Cho/Cr and Cho/NAA ratios are associated with more severe disease and may predict surgical recovery. MRS shows promise as a non-invasive tool for enhancing the diagnosis and management of CSM. Further research is needed to standardize protocols, validate findings in larger cohorts, and integrate MRS into clinical practice.

## Introduction

Cervical spondylotic myelopathy (CSM) is a common acquired condition characterized by the narrowing of the cervical spinal canal at one or more segments, leading to progressive spinal cord injury and functional decline. Although approximately 90–95% of patients with cervical spondylosis are asymptomatic, imaging studies in asymptomatic individuals have reported degenerative changes in one or more cervical levels in up to 95% of men over 60 years of age and 89% of women (1–3).

Currently, magnetic resonance (MR) imaging without contrast is the gold standard for diagnosing CSM. It provides detailed visualization of cervical spine and spinal cord anatomy, reveals the presence and severity of degenerative disease, and assesses the extent of myelopathy. MR imaging also allows evaluation of the spinal cord compression ratio, which is considered an unfavorable prognostic factor for CSM when it is less than 0.436 (4–7).

Poor prognostic indicators on MR imaging typically include low signal intensity on T1-weighted sequences combined with hyperintense lesions on T2-weighted images, suggesting inflammation, edema, or gliosis of the spinal cord (6–10). However, clinical studies have not consistently correlated these MR findings with the patient’s clinical status or prognostic outcomes after treatment (11, 12). Even spinal cord atrophy has not been definitively established as a predictor of clinical outcomes (4, 6, 7).

Magnetic resonance spectroscopy (MRS) is a non-invasive advanced diagnostic technique used to determine the molecular composition of tissue, including phase changes, conformational and configurational alterations, solubility, and diffusion potential. It provides valuable information on cellular biochemistry and neural function (13). Sparse evidence has described decreased N-acetylaspartate (NAA)/creatine (Cr) ratios and abnormal lactate levels in patients with CSM, but there is no consensus on the clinical significance and prognostic relevance of these findings, either pre- or post-operatively (4, 14, 15).

Therefore, in this systematic review, we aim to evaluate the MR spectroscopy findings of the spinal cord in patients with CSM, establish their clinical correlation with validated scales, and assess changes in metabolites after surgical decompression.

## Materials and methods

This systematic review adheres to the standards set by the Cochrane Collaboration and follows the recommendations of the Preferred Reporting Items for Systematic Reviews and Meta-Analyses (PRISMA) 2020 statement (16).

### Search strategy

A comprehensive literature search was conducted using Medical Subject Headings (MeSH) terms and related keywords, accounting for synonyms, acronyms, plurals, and spelling variations. The databases searched included PubMed, OVID, EMBASE, Web of Science, and the Cochrane Central Register of Controlled Trials, covering all records from their inception until June 2023. The search was limited to articles published in English or Spanish, with no time restrictions. An example of the search equation used in PubMed is provided below:

(“cervical vertebrae”[MeSH Terms] OR (“cervical”[All Fields] AND “vertebrae”[All Fields]) OR “cervical vertebrae”[All Fields] OR (“cervical”[All Fields] AND “spine”[All Fields]) OR “cervical spine”[All Fields]) AND (“cervical cord”[MeSH Terms] OR (“cervical”[All Fields] AND “cord”[All Fields]) OR “cervical cord”[All Fields]) AND (“magnetic resonance spectroscopy”[MeSH Terms] OR (“magnetic”[All Fields] AND “resonance”[All Fields] AND “spectroscopy”[All Fields]) OR “magnetic resonance spectroscopy”[All Fields]) AND (“spinal cord compression”[MeSH Terms] OR (“spinal”[All Fields] AND “cord”[All Fields] AND “compression”[All Fields]) OR “spinal cord compression”[All Fields])

### Eligibility criteria and study selection

Studies were considered eligible for inclusion if they met the following criteria:

1. Involved Suspicion of CSM was defined as the presence of symptoms suggestive of myelopathy, such as gait instability, hand dexterity issues, or frequent falls, even if a definitive diagnosis via imaging was not yet established (17, 18).
2. Included a cohort of at least 10 patients.
3. Reported primary data on MR spectroscopy findings of the cervical spine and clinical condition, ideally using validated scales such as the modified Japanese Orthopaedic Association (mJOA) scale, both pre-and post-operatively.

Exclusion criteria were as follows:

- *In vitro* or cadaveric studies, reviews, abstracts, and conference proceedings.
- Studies focusing on causes of cervical myelopathy other than CSM (e.g., tumors, myelodegenerative diseases).
- Studies that did not report pre-operative data.
- Studies that did not comply with any of the inclusion criteria.

All reviewers independently screened the titles and abstracts of the retrieved records. Relevant studies were then reviewed in full text to determine eligibility based on the criteria above. In cases of disagreement, a third reviewer was consulted to reach a consensus.

### Data extraction

Data extraction was performed independently by all reviewers using separate Excel spreadsheets. Extracted data were then cross-checked among reviewers and against the original articles. The following information was collected:

- Study objective.
- Country where the study was conducted.
- Study design.
- Number of patients included.
- Male-to-female ratio.
- Age of participants.
- Clinical symptoms.
- MR spectroscopy protocols and results.
- Follow-up duration.

Any discrepancies in data extraction were resolved through discussion, and if necessary, adjudicated by a third reviewer.

### Levels of evidence

Each study was graded according to the 2011 Oxford Centre for Evidence-Based Medicine (OCEBM) guidelines. Two independent reviewers performed the grading, and any disagreements were resolved by a third reviewer.

### Assessment of heterogeneity

We assessed heterogeneity across the included studies by examining clinical characteristics (e.g., patient populations, interventions), methodological aspects (e.g., study design, data collection methods), and statistical analyses.

### Risk of bias assessment

The risk of bias for each study was evaluated using the Newcastle-Ottawa Scale. Two independent reviewers conducted the assessments, with a third reviewer resolving any discrepancies.

### Qualitative synthesis

A narrative synthesis was performed to summarize the findings of the included studies. This synthesis described the clinical and methodological characteristics, strengths and limitations, quality of evidence, and risk of bias for each study.

## Results

**Figure 1:**
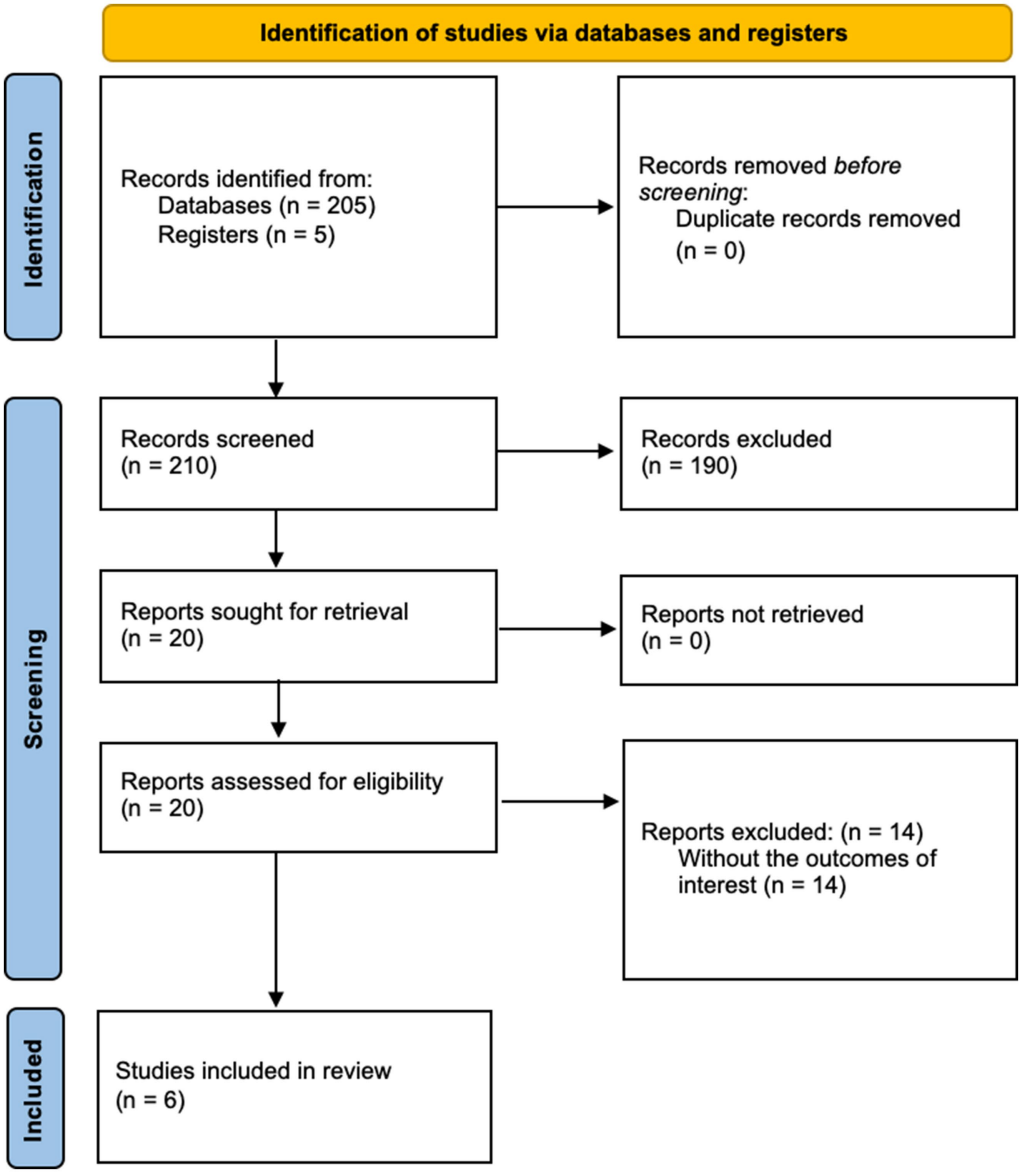
PRISMA flow diagram for study selection process.

The initial search strategy identified a total of 210 records—205 from databases and 5 from manual searches. After removing duplicates, 210 unique studies remained for screening. Title and abstract screening excluded 190 records that did not meet the inclusion criteria. A full-text review was conducted on the remaining 20 studies, of which 14 were excluded due to the absence of reported outcomes directly assessing MR spectroscopy findings correlated with clinical severity (e.g., metabolite ratios or mJOA scores). Ultimately, six studies were included for data extraction, as illustrated in the PRISMA flow diagram (Figure 1).

### Qualitative synthesis

The six included studies were all prospective and published between 2006 and 2017, representing Level 3 evidence according to the Oxford Centre for Evidence-Based Medicine (OCEBM) guidelines. These studies were conducted in Egypt, Turkey, and the United States, involving a total of 123 patients with an average age range of 45.8 to 63 years. Table 1 provides a summary of the main characteristics of the included studies, including patient demographics, study design, and MR spectroscopy details.

**Table 1:** Summary of MR spectroscopy findings in cervical spondylotic myelopathy studies.

Study (Year)	Design	Sample characteristics	Predominant symptoms	MR spectroscopy technical details	Measured outcome
Holly et al., 2013	Case control	*n* = 21, M/F = 12/9, Age = 59	Neck pain, motor dysfunction, paresthesia	Siemens 3 T, PRESS, TR = 2000 ms, TE = 30 ms, 1.72 mL voxel	NAA/Cr and Cho/Cr ratios mJOA pre/post change T2 changes
Taha Ali et al., 2013	Case control	*n* = 24, M/F = 15/9, Age = 53	Not specified	Philips 1.5 T, PRESS, TR = 2000 ms, TE = 36 ms, CHESS for water suppression	NAA/Cr, Cho/Cr ratios Lactate peak
Holly et al., 2017	Cohort	*n* = 16, M/F = 10/6, Age = 63	Gait disturbance, hand paresthesia	Siemens 1.5 T, C2 spinal level, PRESS, TR = 1500/3000 ms, TE = 30 ms	Pre/post mJOA NAA/Cr, Cho/NAA
Kendi et al., 2006	Case control	*n* = 14, M/F = 6/8, Age = 45.8	Neck pain, upper limb pain, paresis	Philips 1.5 T, MOIST water suppression, voxel 5 × 6 × 6 mm	NAA, Choline Creatine, Myoinositol
Ellingson et al., 2015	Case control	*n* = 27, M/F = 13/14, Age = 62	Gait disturbance, neck pain	Siemens 3 T, TR = 2000 ms, TE = 30 ms, 1.72 mL voxel	Cho/NAA, NAA/Cr mJOA correlation
Holly et al., 2009	Case control	*n* = 21, M/F = 11/10, Age = 60	Gait disturbance, hand paresthesia	Siemens 1.5 T, T1 and T2 imaging sequences	NAA/Cr, Cho/Cr - Lactate peaks - mJOA

Cho, Choline; Cr, creatine; Myo, myoinositol; NAA, N-acetyl aspartate; Glix, glicina; Glu, glutamine; Lip, Lipides; Lac, lactate; mJOA, modified Japanese Orthopaedic Association scale.

Common pre-operative symptoms among patients included neck pain, radicular upper-limb pain, paresthesia, and motor impairment. Magnetic Resonance (MR) spectroscopy was performed using a 1.5 Tesla MRI scanner in four studies and a 3 Tesla MRI scanner in two studies. The main metabolites measured were N-acetyl aspartate (NAA), choline (Cho), creatine (Cr), and lactate levels. The studies focused on metabolite ratios such as NAA/Cr, Cho/Cr, and Cho/NAA. Four studies utilized the modified Japanese Orthopedic Association (mJOA) scale to assess symptom severity and functional status.

### Key findings from the studies

The systematic review included six prospective studies that analyzed a total of 123 patients with CSM. These studies provided valuable insights into the metabolic changes detected by MRS and their correlations with clinical severity and outcomes. Symptomatic patients demonstrated consistent reductions in the NAA/Cr ratio, a marker of neuronal loss or dysfunction. For instance, Kendi et al. (19) reported a mean NAA/Cr ratio of 1.46 ± 0.29 in symptomatic patients, which was significantly lower than the 2.01 ± 0.30 observed in healthy controls (*p* < 0.001; Table 2). Similarly, elevated Cho/Cr and Cho/NAA ratios were observed across studies, reflecting increased membrane turnover or gliosis. Salamon et al. (20) highlighted this pattern, noting higher Cho/NAA ratios in symptomatic patients with T2-weighted imaging changes compared to healthy controls (mean ± SD: 1.51 ± 0.21 versus 0.89 ± 0.23, respectively; *p* < 0.001; Table 2).

**Table 2:** Comparative metabolite levels in MR spectroscopy across CSM patient groups.

Study (Year)	Sample size (symptomatic/asymptomatic)	Symptomatic with T2 changes	Symptomatic without T2 changes	Asymptomatic	mJOA	Symptomatic patients with lactate peaks
			NAA / Cr | Cho / Cr | Glix / Cr | (Lip-Lac) / Cr | Mio / Cr | Cho / NAA	NAA / Cr | Cho / Cr | Glix / Cr | (Lip-Lac) / Cr | Mio / Cr | Cho / NAA	NAA / Cr | Cho / Cr | Glix / Cr | (Lip-Lac) / Cr | Mio / Cr | Cho / NAA	mJOA	Count
Ellingson et al., 2015	27	3.95 ± 8.99 | 10.06 ± 36.60 | - | - | - | 1.58 ± 1.82	–	–	–	–
Holly et al., 2013	21/11	1.17 ± 0.42 | 0.49 ± 0.17 | 1.36 ± 0.78 | 0.36 ± 0.38 | 1.31 ± 0.67 | 0.34 ± 0.07	1.27 ± 0.27 | 0.41 ± 0.09 | 2.02 ± 1.44 | 0.27 ± 0.21 | 1.49 ± 0.58 | 0.32 ± 0.05	1.37 ± 0.32 | 0.31 ± 0.08 | 1.68 ± 1.00 | 0.19 ± 0.13 | 1.42 ± 0.57 | 0.25 ± 0.08	14.7–16.6	-
Taha Ali, et al., 2013	24/11	1.34 ± 0.09 | 0.82 ± 0.12 | - | - | - | -	–	1.82 ± 0.08 | 0.75 ± 0.14 | - | - | - | -	–	9
Holly et al., 2009	21 / 13	1.27 ± 0.52 | 0.96 ± 0.24 | - | - | - | -	–	1.83 ± 0.18 | 0.93 ± 0.18 | - | - | - | -	12.3 ± 2.2	7
Kendi et al., 2006	14 (20 measurements)	NAA: ↓19, ↑1 | Creatine: ↑14, ↓6 | Choline: ↓12, ↑8 | Lactate: ↓12, ↑8	–	–	–	–

In addition to these metabolic alterations, several studies reported the presence of lactate peaks in symptomatic patients, which were absent in controls. These findings are indicative of hypoxic or inflammatory injury. For example, Taha Ali et al. (21) observed lactate peaks in 33% of symptomatic cases, suggesting that MRS can detect pathological changes that might be missed by conventional imaging techniques.

The correlation between MRS findings and clinical severity, as measured by the mJOA scores, was a recurring theme in the included studies. Lower NAA/Cr and higher Cho/NAA ratios were strongly associated with reduced mJOA scores, reflecting more severe functional impairment. Ellingson et al. (22) demonstrated a statistically significant inverse relationship between Cho/NAA ratios and mJOA scores (correlation coefficient *r* = −0.65, *p* < 0.01; Table 2). Similarly, Holly et al. (15) found that pre-operative NAA/Cr ratios were predictive of post-operative recovery, with patients showing higher NAA/Cr ratios experiencing greater improvements in mJOA scores following surgical decompression (*p* < 0.05; Table 3).

**Table 3:** Comparison of pre- and post-operative mJOA scores and metabolite ratios in CSM patients.

Study (Year)	Pre-operative mJOA	Post-operative mJOA	NAA/Cr (mean | range)	Cho/Cr (mean | range)	Cho/NAA (mean | range)
Holly et al., 2017	12.1	14.6	1.44 | (0.86–2.24)	0.98 | (0.57–1.45)	0.75 | (0.34–1.45)

Post-operative changes in metabolite ratios further underscored the potential utility of MRS in monitoring recovery. Across the studies, surgical decompression was associated with normalization of metabolite ratios, particularly an increase in NAA/Cr and a decrease in Cho/NAA ratios. Holly et al. (15) reported that patients who exhibited significant post-operative increases in NAA/Cr ratios experienced better functional outcomes, reinforcing the prognostic value of these metabolic markers (Table 3).

Notably, these metabolite changes were consistently observed in symptomatic patients regardless of T2 imaging findings, highlighting the additional value of MRS in detecting biochemical alterations not evident on conventional imaging. The patterns of reduced NAA/Cr, elevated Cho/Cr, and the presence of lactate peaks in symptomatic patients were robust across the included studies, emphasizing the reliability of these findings in characterizing the pathophysiology of CSM.

### Risk of bias

The risk of bias was assessed using the Newcastle-Ottawa Scale. Among the four case–control studies, three were rated as having a low risk of bias, while one was considered to have a moderate risk due to limitations in sample size and participant selection. The cohort study and case series were also rated as having a low risk of bias. Detailed risk of bias evaluations are presented in Tables 4, **5**.

**Table 4:** Newcastle-Ottawa risk of bias assessment for case–control studies in CSM research.

Study, year of publication	Selection				Comparability	Exposure			
	Is the case definition adequate?	Representativeness of the cases	Selection of controls	Definition of controls	Comparability of cases and controls on the basis of the design or analysis	Ascertainment of exposure	Same method of ascertainment for cases and controls	Non-response rate	Quality of evidence
Holly et al., 2013									High
Taha Ali, et al., 2013									High
Holly et al., 2009									High
Kendi et al., 2006									Moderate

Red: High. Orange: Some concerns. Green: Low.

**Table 5:** Newcastle-Ottawa risk of bias assessment for cohort studies in CSM research.

Study, year of publication	Selection				Comparability	Outcome			
	Representativeness of the exposed cohort	Selection of the non-exposed cohort	Ascertainment of exposure	Demonstration that outcome of interest was not present at start of study	Comparability of cohorts on the basis of the design or analysis	Assesment of outcome	Was follow-up long enough for outcomes to occur?	Adequacy of follow up of cohorts	Quality of evidence
Holly et al., 2013									High
Taha Ali, et al., 2013									High

Red: High. Orange: Some concerns. Green: Low.

## Discussion

Cervical spondylotic myelopathy (CSM) is a prevalent condition affecting millions worldwide, often leading to significant morbidity due to motor impairment, upper extremity paresthesia, and neck pain (1–3). Imaging studies indicate that degenerative changes in the cervical spine are present in up to 95% of men and 89% of women over 60 years of age, even among asymptomatic individuals (1, 2). Therefore, advancements in diagnostic tools that facilitate early detection and intervention are crucial for altering the potentially catastrophic course of this disease.

### Significance of MR spectroscopy in CSM

Our systematic review highlights the potential of magnetic resonance (MR) spectroscopy as a non-invasive imaging modality that provides detailed metabolic information about the spinal cord, which is not obtainable through conventional MR imaging. The studies included in this review consistently demonstrated that symptomatic CSM patients exhibit reduced N-acetyl aspartate to creatine (NAA/Cr) ratios and elevated choline to creatine (Cho/Cr) and choline to NAA (Cho/NAA) ratios compared to healthy controls (Table 2) (19–23). Lower NAA/Cr ratios suggest neuronal loss or dysfunction, while elevated Cho levels indicate increased membrane turnover or gliosis—both of which reflect underlying spinal cord pathology (13, 14).

### Correlation with clinical severity and prognosis

A significant finding across the studies is the correlation between metabolite ratios obtained from MR spectroscopy and clinical severity as measured by the modified Japanese Orthopaedic Association (mJOA) scores. Ellingson et al. (2015) reported that higher Cho/NAA ratios were associated with lower mJOA scores, indicating more severe myelopathy (22). Similarly, Holly et al. (15) found that patients with higher pre-operative NAA/Cr ratios experienced greater improvement in mJOA scores following surgical decompression, and post-operative increases in NAA/Cr ratios correlated with better neurological recovery (Table 3). These findings suggest that MR spectroscopy not only reflects the current state of spinal cord injury but may also have prognostic value in predicting surgical outcomes.

### Comparative analysis with other advanced imaging modalities

While conventional MR imaging remains the gold standard for diagnosing CSM, it has limitations in predicting clinical outcomes and correlating imaging findings with symptom severity (4–12). Advanced imaging techniques such as diffusion tensor imaging (DTI) and functional MRI (fMRI) have been explored for their potential to provide additional insights. DTI assesses the integrity of white matter tracts and has shown promise in detecting microstructural changes in the spinal cord (24–28). However, DTI parameters like fractional anisotropy can be influenced by various factors, including edema and inflammation, and may not fully capture the metabolic state of the tissue.

In contrast, MR spectroscopy directly measures biochemical changes within the spinal cord. Recent studies have suggested that combining MR spectroscopy with other imaging modalities may enhance diagnostic accuracy. Wang et al. demonstrated that integrating MR spectroscopy with DTI improved the prediction of surgical outcomes in CSM patients (29). This multimodal approach could offer a more comprehensive assessment of spinal cord pathology.

### Clinical implications and applications

The metabolic changes observed through MRS, such as reduced NAA/Cr and elevated Cho/Cr and Cho/NAA ratios, provide insights into the underlying mechanisms of neuronal loss, gliosis, and hypoxia in CSM. Understanding these processes informs the potential development of targeted therapies aimed at mitigating neuronal damage. For instance, interventions that restore neuronal energy metabolism, enhance neuroprotection, or reduce gliosis could complement surgical decompression and improve functional outcomes.

These findings align with prior research emphasizing the role of metabolic modulation in preserving neuronal function and preventing progressive spinal cord damage (15, 22). Future studies should evaluate whether pharmacological agents, rehabilitation strategies, or other targeted interventions addressing these metabolic pathways could improve long-term recovery and outcomes in CSM patients.

### Pathophysiological insights

The metabolic alterations observed in MR spectroscopy reflect underlying pathophysiological mechanisms in CSM. Decreased NAA levels indicate neuronal loss or dysfunction, which may result from chronic compression and ischemia of the spinal cord. Elevated Cho levels suggest increased cell membrane turnover due to demyelination or gliosis (13). The detection of lactate peaks in symptomatic patients further indicates anaerobic metabolism secondary to hypoxia or inflammatory processes (21, 23).

### Limitations and challenges

Despite the promising findings, several limitations must be acknowledged. First, the number of studies and sample sizes are relatively small, with the included studies involving a total of 123 patients. This small sample size limits the generalizability of the findings and increases the risk of type II errors. Second, there is heterogeneity among the studies in terms of MR spectroscopy protocols, including differences in magnetic field strength, voxel placement, acquisition parameters, and metabolite quantification methods. Such variability makes it challenging to standardize metabolite ratio thresholds and compare results across studies.

Moreover, potential publication bias cannot be excluded. Studies with positive findings are more likely to be published, which may overestimate the effectiveness of MR spectroscopy in CSM. Additionally, the included studies were conducted in specific geographic locations (Egypt, Turkey, and the United States), which may limit the applicability of the results to other populations.

Technical challenges also exist in performing MR spectroscopy of the spinal cord due to its small size, susceptibility to motion artifacts, and proximity to cerebrospinal fluid and vertebral bone. Advanced techniques and specialized equipment are required, which may not be widely available. The interpretation of MR spectroscopy data requires expertise that may not be readily accessible in all clinical settings.

These limitations may affect the interpretation of our results and the conclusions drawn. The heterogeneity among studies and small sample sizes necessitate caution when generalizing these findings to broader clinical practice. Larger, multicenter studies with standardized MR spectroscopy protocols are needed to validate these preliminary findings.

### Economic and practical considerations

The cost and availability of MR spectroscopy are practical considerations that could limit its widespread adoption. High-field MR scanners and specialized coils are expensive, and the procedure is time-consuming compared to standard MR imaging. Cost-effectiveness studies are needed to determine whether the benefits of incorporating MR spectroscopy into routine clinical practice justify the additional resources required.

### Future research directions

To fully realize the potential of MR spectroscopy in CSM, future research should focus on:

- Standardization of protocols: developing standardized acquisition and analysis protocols to improve consistency across studies and enable the establishment of normative data for metabolite ratios.
- Large-scale multicenter studies: conducting larger studies with diverse patient populations to validate the prognostic value of MR spectroscopy findings and assess their generalizability.
- Integration with other modalities: exploring multimodal imaging approaches that combine MR spectroscopy with DTI, fMRI, or other techniques to enhance diagnostic accuracy and prognostic capabilities.
- Technological advancements: investing in technological improvements to overcome current technical challenges, such as motion correction algorithms and advanced coils designed for spinal cord imaging.
- Clinical trials: designing prospective clinical trials to assess the impact of MR spectroscopy-guided decision-making on patient outcomes.

## Conclusion

In conclusion, MR spectroscopy offers a promising avenue for improving the diagnosis, prognostication, and management of cervical spondylotic myelopathy. By providing metabolic information that correlates with clinical severity and functional outcomes, MR spectroscopy has the potential to enhance patient care through earlier detection and more personalized treatment strategies. However, due to limitations such as small sample sizes, study heterogeneity, and potential publication bias, further research is necessary to overcome current challenges, standardize methodologies, and validate the clinical utility of MR spectroscopy in larger patient cohorts. Future studies should focus on integrating MR spectroscopy with other advanced imaging modalities and conducting large-scale, multicenter trials to establish its role in routine clinical practice.

## Data Availability

The raw data supporting the conclusions of this article will be made available by the authors, without undue reservation.

## References

[1] Tsuji T Fujiwara H Nishiwaki Y Daimon K Okada E Nojiri K . Modic changes in the cervical spine: prospective 20-year follow-up study in asymptomatic subjects. J Orthop Sci. (2019) 24:612–7. doi: 10.1016/j.jos.2018.12.015, PMID: 30642726

[2] Wu J-C Ko C-C Yen Y-S Huang W-C Chen Y-C Liu L . Epidemiology of cervical spondylotic myelopathy and its risk of causing spinal cord injury: a national cohort study. Neurosurg Focus. (2013) 35:E10. doi: 10.3171/2013.4.focus13122, PMID: 23815246

[3] Bajamal AH Kim S-H Arifianto MR Faris M Subagio EA Roitberg B . Posterior surgical techniques for cervical Spondylotic myelopathy: WFNS spine committee recommendations. Neurospine. (2019) 16:421–34. doi: 10.14245/ns.1938274.137, PMID: 31607074 PMC6790723

[4] Zileli M Maheshwari S Kale SS Garg K Menon SK Parthiban J. Outcome measures and variables affecting prognosis of cervical Spondylotic myelopathy: WFNS spine committee recommendations. Neurospine. (2019) 16:435–47. doi: 10.14245/ns.1938196.098, PMID: 31607075 PMC6790741

[5] Li X-Y Lu S-B Sun X-Y Kong C Guo M-C Sun S-Y . Clinical and magnetic resonance imaging predictors of the surgical outcomes of patients with cervical spondylotic myelopathy. Clin Neurol Neurosurg. (2018) 174:137–43. doi: 10.1016/j.clineuro.2018.09.003, PMID: 30241007

[6] Matsuyama Y Kawakami N Yanase M Yoshihara H Ishiguro N Kameyama T . Cervical myelopathy due to OPLL. J Spinal Disord Tech. (2004) 17:401–4. doi: 10.1097/01.bsd.0000112087.85112.86, PMID: 15385880

[7] Ahn J-S Lee J-K Kim B-K. Prognostic factors that affect the surgical outcome of the Laminoplasty in cervical Spondylotic myelopathy. Clin Orthop Surg. (2009) 2:98–104. doi: 10.4055/cios.2010.2.2.98, PMID: 20514267 PMC2867205

[8] You JY Lee JW Lee E Lee GY Yeom JS Kang HS. MR classification system based on axial images for cervical compressive myelopathy. Radiology. (2015) 276:553–61. doi: 10.1148/radiol.2015142384, PMID: 25906184

[9] Dokai T Nagashima H Nanjo Y Tanida A Teshima R. Surgical outcomes and prognostic factors of cervical spondylotic myelopathy in diabetic patients. Arch Orthop Trauma Surg. (2012) 132:577–82. doi: 10.1007/s00402-011-1449-4, PMID: 22203056

[10] Nouri A Martin AR Mikulis D Fehlings MG. Magnetic resonance imaging assessment of degenerative cervical myelopathy: a review of structural changes and measurement techniques. Neurosurg Focus. (2016) 40:E5. doi: 10.3171/2016.3.focus1667, PMID: 27246488

[11] Yukawa Y Kato F Yoshihara H Yanase M Ito K. MR T2 image classification in cervical compression myelopathy. Spine. (2007) 32:1675–8. doi: 10.1097/brs.0b013e318074d62e, PMID: 17621217

[12] Yukawa Y Kato F Ito K Horie Y Hida T Machino M . Postoperative changes in spinal cord signal intensity in patients with cervical compression myelopathy: comparison between preoperative and postoperative magnetic resonance images. J Neurosurg Spine. (2008) 8:524–8. doi: 10.3171/spi/2008/8/6/524, PMID: 18518672

[13] Krishnan VV. Molecular thermodynamics using nuclear magnetic resonance (NMR) spectroscopy. Inventions. (2019) 4:13. doi: 10.3390/inventions4010013, PMID: 31123720 PMC6528671

[14] Martin AR Aleksanderek I Cohen-Adad J Tarmohamed Z Tetreault L Smith N . Translating state-of-the-art spinal cord MRI techniques to clinical use: a systematic review of clinical studies utilizing DTI, MT, MWF, MRS, and fMRI. NeuroImage Clin. (2016) 10:192–238. doi: 10.1016/j.nicl.2015.11.019, PMID: 26862478 PMC4708075

[15] Holly LT Ellingson BM Salamon N. Metabolic imaging using proton magnetic spectroscopy as a predictor of outcome after surgery for cervical Spondylotic myelopathy. Clin Spine Surg. (2017) 30:E615–9. doi: 10.1097/bsd.0000000000000248, PMID: 28525487 PMC4510035

[16] Page MJ McKenzie JE Bossuyt PM Boutron I Hoffmann TC Mulrow CD . The PRISMA 2020 statement: an updated guideline for reporting systematic reviews. BMJ. (2021) 372:n71. doi: 10.1136/bmj.n71, PMID: 33782057 PMC8005924

[17] Nurjck S. The pathogenesis of the spinal cord disorder associated with cervical spondylosis. Brain. (1972) 95:87–100. doi: 10.1093/brain/95.1.87 5023093

[18] Tetreault L Kopjar B Nouri A Arnold P Barbagallo G Bartels R . The modified Japanese Orthopaedic association scale: establishing criteria for mild, moderate and severe impairment in patients with degenerative cervical myelopathy. Eur Spine J. (2017) 26:78–84. doi: 10.1007/s00586-016-4660-8, PMID: 27342612

[19] Kendi ATK Bademci G Kara SA Keskil S Erdal HH. MR spectroscopy of cervical spinal cord in patients with clinical and/or Electrophysiologic signs of spinal cord compression. Neuroradiol J. (2006) 19:134–9. doi: 10.1177/197140090601900118

[20] Salamon N Ellingson BM Nagarajan R Gebara N Thomas A Holly LT. Proton magnetic resonance spectroscopy of human cervical spondylosis at 3T. Spinal Cord. (2013) 51:558–63. doi: 10.1038/sc.2013.31 23588574 PMC3703492

[21] Ali TFT Badawy AE. Feasibility of 1H-MR spectroscopy in evaluation of cervical spondylotic myelopathy. Egypt J Radiol Nucl Med. (2013) 44:93–9. doi: 10.1016/j.ejrnm.2012.11.001, PMID: 39816934

[22] Ellingson BM Salamon N Hardy AJ Holly LT. Prediction of neurological impairment in cervical Spondylotic myelopathy using a combination of diffusion MRI and proton MR spectroscopy. PLoS One. (2015) 10:e0139451. doi: 10.1371/journal.pone.0139451, PMID: 26431174 PMC4592013

[23] Holly LT Freitas B McArthur DL Salamon N. Proton magnetic resonance spectroscopy to evaluate spinal cord axonal injury in cervical spondylotic myelopathy: laboratory investigation. J Neurosurg Spine. (2009) 10:194–200. doi: 10.3171/2008.12.spine08367, PMID: 19320577

[24] Nilsson M Lätt J Ståhlberg F Van WD Hagslätt H. The importance of axonal undulation in diffusion MR measurements: a Monte Carlo simulation study. NMR Biomed. (2012) 25:795–805. doi: 10.1002/nbm.1795, PMID: 22020832

[25] Facon D Ozanne A Fillard P Lepeintre J-F Tournoux-Facon C Ducreux D. MR diffusion tensor imaging and fiber tracking in spinal cord compression. AJNR Am J Neuroradiol. (2005) 26:1587–94. PMID: 15956535 PMC8149058

[26] Cheung MM Li DTH Hui ES Fan S Ding AY Hu Y . In vivo diffusion tensor imaging of chronic spinal cord compression in rat model. Annu Int Conf IEEE Eng Med Biol Soc. (2009) 2009:2715–8. doi: 10.1109/iembs.2009.5333389, PMID: 19964039

[27] Jones JGA Cen SY Lebel RM Hsieh PC Law M. Diffusion tensor imaging correlates with the clinical assessment of disease severity in cervical Spondylotic myelopathy and predicts outcome following surgery. Am J Neuroradiol. (2013) 34:471–8. doi: 10.3174/ajnr.a3199, PMID: 22821918 PMC7965104

[28] Mamata H Jolesz FA Maier SE. Apparent diffusion coefficient and fractional anisotropy in spinal cord: age and cervical spondylosis–related changes. J Magn Reson Imaging. (2005) 22:38–43. doi: 10.1002/jmri.20357, PMID: 15971186

[29] Wang X Tian X Zhang Y Zhao B Wang N Gao T . Predictive value of dynamic diffusion tensor imaging for surgical outcomes in patients with cervical spondylotic myelopathy. BMC Méd Imaging. (2024) 24:260. doi: 10.1186/s12880-024-01428-9, PMID: 39354411 PMC11445957

